# The Rise of Clinical Pathology

**Published:** 1912-12-21

**Authors:** 


### THE RISE OF CLINICAL PATHOLOGY.

In the collection of charming and brilliant essays published a year or two ago under the title of *The Corner of Harley Street* some genial fun is poked at a certain physician who once informed a class of students that he looked forward to the day when he should be met at the doors of the hospital by a chemist, a bacteriologist, and a pathologist. To these subordinates, the physician remarked, he would assign their respective parts in the elucidation of each case of disease; and he pictured himself, according to *Peter Harding*, as co-ordinating their reports and thus making the diagnosis of the disease. The incident is alleged by those who know the London Hospital to be founded upon fact, and the lesson which it teaches is much the same as that which is enforced in the article printed in another column this week (page 319). As *Peter Harding* was acute enough to see, there is a definite danger lest the extraordinary aids which clinical pathology can render to clinical medicine and surgery should obsess the practitioners of the latter, and this danger manifests itself in two distinct forms. In the first place there is a risk that some really conscientious and painstaking physicians, anxious to make the fullest use of modern advances, should neglect to some extent the study of physical signs and symptoms in their preoccupation with what one may call laboratory methods of diagnosis. These physicians have, as a rule, had personal training in clinical pathology, and their fault is merely the result of a natural predilection for test-tubes and microscopes as opposed to palpation and percussion and the rest. They are not perhaps a numerous class, and undeniably some of them have contributed to the progress of modern medicine. The other class is that which the essayist so justly pilloried; men who, without first-hand knowledge of the possibilities and the limitations of laboratory methods, see in them a convenient means of posing as great clinicians, while at the same time actually shirking clinical difficulties and lessening their responsibility.

The more ambitious of the clinical pathologists are demanding that they should consult direct with the man in charge of the case, instead of merely answering "Yes" or "No" to specific questions on a pink card or a blue card addressed to them by a physician who may be ignorant of the true import of the answer and very often vitiates it by errors of the kind which are instanced in the article on p. 319. This claim is in our opinion a just and necessary one, and, though the pseudo-scientific and pretentious brand of clinician will probably resent it, in the end it will have to be conceded. The only other solution of the difficulty is that advocated by our contributor at the close of his article, in which he proposes that each hospital should possess a very good clinical laboratory manned by first-rate unqualified assistants, and that therein the physicians and surgeons should themselves superintend the work in connection with their own cases. The suggestion would doubtless prove very valuable if its working were practicable, but it is to be feared that the threefold claims of private practice, teaching, and ward work render it impossible for the vast majority of those who hold staff appointments at hospitals to find the necessary time for laboratory work.

In addition, we cannot help thinking that research would suffer by such a system. Experience shows that the men who make discoveries are, as a rule, specialists; the man who devotes his whole time to clinical pathology is a specialist, and should be recognised and given standing as such. He should be on equal terms with the physician and the surgeon, free to confer with them on an equal footing instead of taking orders from them as a subordinate. At the same time the responsibility for diagnosis and treatment should remain with the physician (or surgeon), who should be neither too proud to receive instruction in matters with which he is not fully conversant from the clinical pathologist, nor too prone to neglect his own proper functions by reliance exclusively upon indices and reactions.

